# Is there an association between long-term antibiotics for acne and subsequent infection sequelae and antimicrobial resistance? A systematic review

**DOI:** 10.3399/BJGPO.2020.0181

**Published:** 2021-04-07

**Authors:** Ketaki Bhate, Liang-Yu Lin, John S Barbieri, Clémence Leyrat, Susan Hopkins, Richard Stabler, Laura Shallcross, Liam Smeeth, Nick Francis, Rohini Mathur, Sinéad M Langan, Sarah-Jo Sinnott

**Affiliations:** 1 Faculty of Epidemiology and Population Health, London School of Hygiene and Tropical Medicine, London, UK; 2 University of Pennsylvania, Perelman School of Medicine, Philadelphia, PA, USA; 3 AMR Division, Public Health England, London, UK; 4 Faculty of Infectious and Tropical Diseases, London School of Hygiene and Tropical Medicine, London, UK; 5 Faculty of Population Health Sciences, University College London, London, UK; 6 School of Primary Care, Population Sciences and Medical Education, University of Southampton, Southampton, UK

**Keywords:** acne vulgaris, antibiotic, antimicrobial resistance, tetracycline, macrolides, dihydrofolate reductase inhibitor

## Abstract

**Background:**

Antimicrobial resistance (AMR) is a global health priority. Acne vulgaris is a common skin condition for which antibiotic use ranges from a few months to years of daily exposure.

**Aim:**

To systemically search for and synthesise evidence on the risk of treatment-resistant infections, and other evidence of AMR, following long-term oral antibiotic use for acne.

**Design & setting:**

In this systematic review, a literature search was carried out using the databases Embase, MEDLINE, Cochrane, and Web of Science. They were searched using MeSH, Emtree, or other relevant terms, and followed a pre-registered protocol.

**Method:**

Search strategies were developed with a librarian and undertaken in July 2019. All searches date from database inception. The primary outcome was antibiotic treatment failure or infection caused by a resistant organism. Secondary outcomes included detection of resistant organisms without an infection, rate of infection, or changes to flora.

**Results:**

A total of 6996 records were identified. Seventy-three full-text articles were shortlisted for full review, of which five were included. Two investigated rates of infection, and three resistance or changes to microbial flora. Three studies had 35 or fewer participants (range 20–118 496). Three studies had a serious or high risk of bias, one moderate, and one a low risk of bias. Weak evidence was found for an association between antibiotic use for acne and subsequent increased rates of upper respiratory tract infections and pharyngitis.

**Conclusion:**

There is a lack of high quality evidence on the relationship between oral antibiotics for acne treatment and subsequent AMR sequelae. This needs to be urgently addressed with rigorously conducted studies.

## How this fits in

AMR is a global threat and the prolonged use of antibiotics in the treatment of skin conditions may contribute to this burden. Long-term oral antibiotics are frequently used to treat acne in relatively well, young adult populations. This review has highlighted the dearth of high quality studies on the implications of long-term oral antibiotic use on infectious or AMR sequelae. It is not understood how the long-term use of oral antibiotics for acne affects the subsequent rate of infections, changes to microbiota, or AMR. This systematic review has highlighted an urgent need for rigorous, well-conducted studies investigating the relationship between long-term antibiotics for acne and AMR.

## Introduction

The World Health Organization has declared the threat of AMR a most urgent crisis.^1^ Currently, approximately 700 000 people die per year as a result of AMR and a report predicted that there will be 50 million deaths per year as a result of AMR by 2050, with a total cumulative cost to lost global production of 100 trillion USD.^2^ Acne vulgaris is a chronic, inflammatory skin disorder, predominantly of adolescence. It affects 80–100% of adolescents, and 20% have moderate to severe acne.^3^ Topical and oral antibiotics are commonly prescribed in the treatment of acne. Although there is conflicting information in international acne guidelines, they generally recommend treatment with an oral or topical antibiotic for 3–6 months.^4–9^ Tetracyclines and macrolides are the two most common oral antibiotic classes prescribed for people with acne in UK primary care.^4^

The overuse of antibiotics is a cause of AMR. Exposures to antibiotics selects for bacteria with spontaneous or acquired mechanisms of resistance. In turn, commensal bacteria also develop and acquire mechanisms to resist the effects of antibiotics, which may give rise to invasive infection. While it is understood that acne is not an infectious disease and the pathophysiology of acne is multifactorial, with *Cutibacterium acnes* implied in one step in the development of an acne lesion, several studies have shown topical antibiotics for acne leads to resistant *C. acnes*.^10–14^ Less is known about whether antibiotic treatment for acne impacts on bacterial flora at other sites. Despite this, oral antibiotics are considered to have anti-inflammatory effects, and their short-term efficacy ensures continued use, alongside other treatments used for acne such as isotretinoin.^15,16^ Given the potential relationship between exposure to antibiotics and AMR, this practice may not be sustainable.^17^

Antimicrobial stewardship, a framework employed to ensure the judicious use of antibiotics, is effective for other infections in other settings;^18^ however, to ensure its implementation in acne treatment, evidence is needed to show that using antibiotics for acne increases future infective episodes and resistance sequelae. Until this evidence is obtained, there will be little impetus to change clinical practice.^19^

The question of whether antibiotics for acne contribute towards AMR is an evidence gap that needs to be urgently addressed.^20^ This study aims to address this gap by systematically reviewing published evidence on the association between long-term use of oral antibiotics for acne and subsequent risk of antibiotic treatment failure, infection caused by a resistant organism, or other evidence of AMR.

## Method

The review protocol was registered on PROSPERO on 8 of April 2019 before the literature search (www.crd.york.ac.uk/PROSPERO) and is published in *BMJ Open*.^21^ PRISMA (Preferred Reporting Items for Systematic Reviews and Meta-analyses) and RECORD (Reporting of studies Conducted Using Observational Routinely collected Data) guidance was followed.^22^

### Literature search strategy

The databases Embase, MEDLINE, Cochrane, and Web of Science were searched. Search terms were developed by finding keywords from relevant articles and by running pilot searches. Searches were developed alongside a librarian to ensure completeness. To keep the searches as broad as possible the ‘explode’ function on Ovid was used. The search strategy was reviewed by all authors. The final searches were undertaken by the lead author who has medical and search training. Searches were undertaken in July 2019 from inception of the databases.

### Inclusion and exclusion criteria

The review included randomised controlled trials, and both cohort and case–control observational studies. Conference abstracts were included if the full article was unpublished but the full manuscript could be obtained from the authors. Studies were included if they met the above criteria in addition to the following criteria:

- The study population included participants aged ≥8 years with acne, in any healthcare setting.
- The study investigated oral antibiotics prescribed for acne, for a minimum of 28 days of daily dosing.
- The comparison group included people who have not been treated with oral antibiotics for acne (or the general population).
- Studies where outcomes met the primary outcome of antibiotic treatment failure or infection caused by a resistant organism, or the secondary outcome of the detection of resistant organisms without an infection, rate of infection, or changes to bacterial flora. Any measure including proxy measures were used.

Ecological studies and studies that did not assess temporality or looked at specific subtypes of acne (for example, acne fulminans) were excluded. Unpublished, ongoing, and studies in grey literature were excluded. Studies that only looked at AMR of *C. acnes* or those including people aged <8 years were excluded, as acne vulgaris is unlikely to present in children aged <8 years and tetracyclines are not recommended in younger children.

### Exposure and comparator

The exposure was at least 28 days of continuous daily doses of antibiotics for acne. This duration was chosen as 28 days is the usual minimum duration of therapy for acne and it was more likely to distinguish between people receiving antibiotics for acne and those receiving short-course antibiotics for an acute infection. Topical antibiotics were excluded as these are less likely to have an effect at sites other than the skin where they are applied. The comparator group included people with acne who were not treated with oral antibiotics or the general population.

### Outcome

The primary outcome was antibiotic treatment failure (insufficient clinical improvement following treatment of an infection with an antibiotic), or any infection caused by a resistant organism. The secondary outcome was the detection of resistant organisms without a clinical infection, rate of infection, or changes to flora. This included: any measure of AMR, for example, laboratory measures (such as a raised C-reactive protein [an inflammatory marker, which if raised may support the diagnosis of a persistent infection despite prior treatment with an antibiotic or it can be used to monitor antibiotic treatment response to infection] or positive culture in the case of a subsequent resistant infection at any body site); patient observations (such as an elevated temperature and/or pulse rate [which may indicate an infection and could represent antibiotic treatment failure if persistent after treatment with an antibiotic]); or proxy measures that may have been used in epidemiological studies, for example, difficult-to-treat infections. Antibiotic treatment failure is a proxy for AMR. The outcome could occur at any time point after at least 28 days of continuous oral antibiotic exposure for acne. Outcome measures were developed a priori.

### Eligibility assessment and data extraction

Covidence, an online literature review data management programme, was used to facilitate the systematic review process.^23^ All titles and abstracts were uploaded to Covidence. Duplicates were removed and three reviewers — KB, LYL, and JB — independently screened the search results based on title and abstract. Each title and/or abstract needed two votes to undergo full-text review. Conflicts were resolved by the involvement of a fourth reviewer not involved in the screening process, SML.

Full-text articles were assessed independently by the same reviewers. The extraction of the first included record was piloted by all reviewers and discrepancies were discussed. The Cochrane Risk of Bias 2 (RoB 2) tool was used to assess the risk of bias in randomised studies and Risk of Bias in Non-randomised Studies - of Interventions (ROBINS-I) tool was used to assess the risk of bias in non-randomised studies.^24,25^ Grading of Recommendations, Assessment, Development and Evaluations (GRADE) was used to make an overall assessment of the quality of evidence.^26^ Pairs of reviewers made independent assessments of the risk of bias.

## Results

**Figure 1.**
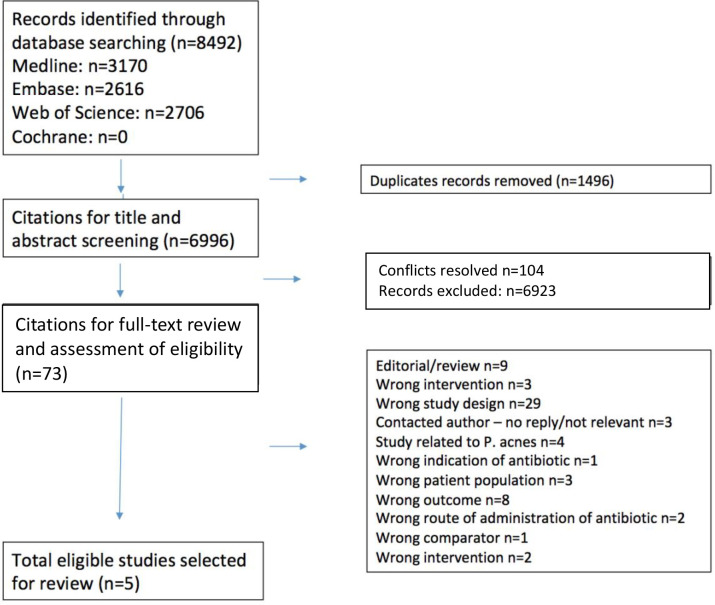
Flow diagram of study selection

A total of 6996 records were identified for title and abstract screening after de-duplication (Figure 1). Of these, 73 full-text articles were shortlisted for full-text review. The full-text of one study could not be obtained despite contacting library repositories in both the UK and US as well as contacting authors; this study was therefore excluded. Overall, five studies were included in the systematic review.^27–31^ The reasons the full-text articles were excluded are in Supplemetary Appendix A. The characteristics of the included studies are summarised in supplementary Table 1, and study results, risk of bias, and overall GRADE assessment are summarised in supplementary Table 2 and Tables 1–3.

**Table 1.** Risk of bias summary showing judgments about each risk of bias domain in ROBINS-I and overall bias assessment across all domains

	**Domain 1: Bias owing to confounding**	**Domain 2: Bias in selection of participants into the study**	**Domain 3: Bias in classification of interventions**	**Domain 4 ITT: Bias owing to deviations from intended interventions: effects of assignment to intervention**
**First author, publication year**	**LYL**	**JB**	**KB**	**LYL**	**JB**	**KB**	**LYL**	**JB**	**KB**	**LYL**	**JB**	**KB**
Margolis 2005^27^		Low	Low		Low	Low		Low	Low		NI	NI
Margolis 2012^28^		Low	Low		Moderate	Moderate		Low	Low		NI	NI
Basak 2013^29^	Critical	Critical		Moderate	Moderate		Low	Low		NI	NI	
Adams 1985^31^	Critical	Critical		NI	NI		Low	Low		NI	NI	

**Domain 4: Bias owing to deviations from intended interventions: effect of starting and adhering to intervention**			**Domain 5: Bias owing to missing data**			**Domain 6: Bias in measurement of outcomes**			**Domain 7: Bias in selection of the reported result**			**Overall bias assessment across all domains**		
**LYL**	**JB**	**KB**	**LYL**	**JB**	**KB**	**LYL**	**JB**	**KB**	**LYL**	**JB**	**KB**	**LYL**	**JB**	**KB**
	NI	NI		Low	Low		Low	Low		Low	Low		Low	Low
	NI	NI		Moderate	Moderate		Serious	Serious		Low	Low		Moderate	Moderate
NI	NI		Low	Low		Low	Low		Serious	Serious		Serious	Serious	
NI	NI		NI	NI		Moderate	Moderate		Moderate	Moderate		Serious	Serious	

Authors: LYL, JB, KB. ITT = intention-to-treat. NI = no information. ROBINS-I = Risk of Bias in Non-randomised Studies - of Interventions.

**Table 2.** The Risk of Bias 2 (RoB 2) assessment for randomised controlled trial^30^

**Borglund *et al*^30^ RoB 2**	LYL	KB
Domain 1 Randomisation process	High	High
Domain 2 Deviations from intended interventions	High	High
Domain 3 Missing outcome data	Low	Low
Domain 4 Measurement of the outcome	Some concerns	Some concerns
Domain 5 Selection of the reported results	Some concerns	Some concerns
Overall risk of bias	High	High

Authors: LYL, KB.

**Table 3.** Summary of findings (GRADE assessment of quality of evidence)

**Summary of findings**
Number of studies	Study design	Risk of bias	Inconsistency	Indirectness	Imprecision	Other considerations	Number of patients	Quality
**Rate of infection**								
2	Cohort	Not serious	Not serious	Not Serious	Serious	No: publication bias, large effect, plausible confounding, dose response gradient	Intervention total: 79 807, Control total: 33 792	⨁⨁LOW a,b
**Detection of resistant organisms without an infection or changes to flora or microbiota**								
3	1 RCT and 2 cohort studies	Serious	Not serious	Not serious	Serious	No: publication bias, large effect, plausible confounding, dose response gradient	Intervention total: 36, Control total: 45	⨁VERY LOW c,d,e,f,d,g

**Explanations**
a. Selection bias: students selected from one university campus.
b. Imprecise estimates: wide 95% confidence intervals.
c. Selection bias: patients not randomised to treatment.
d. Confounding factors not reported or incorporated in analysis.
e. Follow-up inconsistent between treatment groups.
f. Confidence intervals not reported and small sample size.
g. No 95% confidence intervals reported: predominantly numbers and percentages reported.

GRADE = Grading of Recommendations Assessment, Development and Evaluation; RCT = randomised controlled trial.

### Study characteristics

None of the five included studies measured the primary outcomes; three studies investigated the carriage or AMR bacteria using bacterial culture methods, and two studies investigated the rate of infection following antibiotics for acne. Only one study was a randomised controlled trial;^30^ the remaining four were all cohort studies, two of which were undertaken involving patients solely in the UK, and one of those used routinely collected medical records from UK general practice. All studies were from high or upper-middle income countries (three studies from the UK, one from Sweden, and one from Turkey). Study sizes ranged from 20–118 496 participants, and three studies had 35 or fewer included individuals. The mean age of study participants ranged from 17.6–21.7 years (age range 15–38 years).

Given the heterogeneity of included studies, particularly with regard to outcomes, it was not possible to perform a meta-analysis. Therefore, the results of this systematic review are reported narratively.

Borglund *et al* ^30^ investigated changes in the quantity and resistance patterns of skin and intestinal flora in a randomised controlled trial comparing topical clindamycin 1% along with a tablet placebo, and tetracycline 250 mg twice a day orally along with a topical placebo.^30^ The authors reported pronounced reductions in the numbers of streptococci, enterococci, fusobacteria, and enterobacteria in the colon during the treatment period with oral tetracycline and, in particular, new colonisation with tetracycline-resistant strains was noted. The flora normalised to pre-treatment levels 8 weeks after treatment was stopped. Resistance to tetracycline during treatment was seen in 40% of the staphylococcal and enterococcal isolates from the skin.

Two of the studies Margolis *et al* 2005^27^ and Margolis *et al* 2012^28^ investigated the rate of infections following the use of antibiotics for acne. The first used routinely collected electronic health records from the UK (Clinical Practice Research Datalink, formerly General Practice Research Datalink) (*n* = 118 496) to evaluate the association between oral antibiotics prescribed for acne and subsequent upper respiratory tract infections (URTI) and urinary tract infections (UTI).^27^ The authors identified statistically significant associations between being prescribed a long-term oral antibiotic for acne (*n* = 197) and having a subsequent consultation coded for a URTI (odds ratio [OR] = 2.75 (95% confidence interval [CI] = 2.37 to 3.18) or UTI (in women; OR = 1.87 [95% CI = 1.38 to 2.53]; information received via communication with authors [numbers of UTI in men too small for analysis]). The number of individuals with a UTI diagnosis who had received an oral antibiotic for their acne was not reported.

The second study by Margolis *et al* was a cohort study in 2012 (*n* = 579), which investigated the risk of developing pharyngitis in students with acne receiving antibiotic treatment who were based on one university campus in North America.^28^ Thirty-six (6.2%) individuals took an oral antibiotic for their acne. Four out of 36 (11.3%) of those taking an antibiotic for acne reported an episode of pharyngitis compared with 18 out of 543 (3.3%) of those not taking an antibiotic for their acne. The OR associating oral antibiotic use with pharyngitis was 4.34 (95% CI = 1.51 to 12.47) using mixed model multivariable regression.

The final two studies investigated changing resistance patterns among flora following exposure to oral antibiotics for acne. Adams *et al* studied the changing pattern of bowel flora resistance in 26 individuals comprising patients with acne receiving oral erythromycin (*n* = 6) and tetracycline (*n* = 5) and family members living in the same household as the patient with acne.^31^ Patients who had received tetracycline for acne and their relatives developed greater numbers of tetracycline *Escherichia coli* resistant isolates. Conversely, the numbers of erythromycin-resistant *E. coli* isolates decreased in acne patients receiving an antibiotic for acne but increased in their relatives.

The other study aimed to investigate changes in the microbial flora of the nose, oropharynx, and faeces following use of systemic isotretinoin (*n* = 20) and oral antibiotic therapy (*n* = 15).^29^ The authors described it as a randomised controlled trial, however, patients were placed into treatment groups based on acne severity with no description of any random element to treatment allocation. The methods stated that logistic regression was used in analyses, however, no odds ratios were presented. The study reported that antibiotics caused less differentiation (which authors defined as the isolation of *Salmonella* spp., *Shigella* spp., *Pseudomonas aeruingosa* and extended-spectrum beta-lactamase [ESBL] gram negative bacilli) of microbial flora compared with isotretinoin at all the cultured sites.

## Discussion

### Summary

This systematic review found five studies that met the inclusion criteria. All studies investigated secondary outcomes: the detection of resistant organisms without an infection or the rate of infection. No studies in the review addressed the primary outcome of antibiotic treatment failure or infection caused by a resistant organism. Overall, across all outcomes, low or very low quality of evidence was found supporting long-term oral antibiotics for acne being associated with infectious outcomes or AMR (Table 3).

The mechanisms for how *C. acnes* (the bacterium pathophysiologically implicated in the formation of an acne lesion) becomes resistant to topical antibiotics used to treat acne are well described, but oral antibiotic treatments for acne are distributed throughout the body, and the impact of their use on the spread of AMR and risk of treatment-resistant infections is not fully understood.^32,33^ There are reviews aiming to summarise the evidence of AMR secondary to antibiotics for acne; however, this is the first systematic review to the authors' knowledge that aims to address infectious outcomes and resistance of flora other than *C. acnes* as a result of oral antibiotics for acne.

### Strengths and limitations

Strengths of the systematic review included: following a pre-specified protocol published on PROSPERO and *BMJ Open;* designing and reporting the review following PRISMA guidance; undertaking a comprehensive search developed in collaboration with a librarian; having no language or time limits; and completing a full bias risk assessment and reporting the overall quality of evidence using GRADE. In addition, the screening process was undertaken by medical healthcare professionals with epidemiological training.^22^ Limitations included not searching the grey literature, and the lack of studies from developing countries where antibiotics may be used for acne and may be bought over the counter.

### Implications for research and practice

This review has highlighted the dearth of high quality scientific research on the implications of long-term oral antibiotic use for acne on infectious or AMR sequelae. The impact that use of oral antibiotics for acne has on microbial resistance in commensal organisms and difficult-to-treat infections caused by organisms resistant to common antibiotics remains unclear. The degree to which cross-resistance to antibiotic classes other than the one prescribed for acne is also unclear.^34,35^ Given the predicted impact of AMR on death rates — in the order of one death every 3 seconds by 2050 — and the widespread use of long-term oral antibiotics for acne in a relatively healthy, young population,^2^ it is imperative to understand how these antibiotics may contribute to the burden of AMR with high quality prospective studies, so that practice can be modified if needed.

## Registration number

The review protocol was registered on PROSPERO on 8 April 2019 prior to literature search, (CRD42019121738 – www.crd.york.ac.uk/PROSPERO)
